# Evaluation of endolaser thermoablation of the small saphenous vein under local anesthesia

**DOI:** 10.1590/1677-5449.200215

**Published:** 2021-08-02

**Authors:** Filipe Cézar Bertassoni de Souza, Walter Jr. Boim de Araujo, Adriana Buechner de Freitas Brandao, Camila de Almeida Mazzoni, Fabiano Luiz Erzinger, Filipe Carlos Caron, Viviane Gomes Milgioransa Ruggeri

**Affiliations:** 1 Hospital Angelina Caron, Campina Grande do Sul, PR, Brasil.; 2 Instituto da Circulação - Excelência em Angiologia, Cirurgia Vascular e Endovascular, Curitiba, PR, Brasil.

**Keywords:** varicose veins, endolaser thermoablation, chronic venous disease, small saphenous vein, local anesthesia

## Abstract

**Background:**

The small saphenous vein (SSV) is affected in 15% of chronic venous insufficiency (CVI) cases. Conventional surgery is the standard technique for treatment of SSV insufficiency, but sural nerve injury is a complication of great concern. Endovenous laser ablation is a surgical technique for treatment of CVI that is considered likely to reduce morbidity and mortality.

**Objectives:**

To evaluate patients with CVI undergoing endovenous laser ablation of the SSV at least 30 days after the procedure.

**Methods:**

We analyzed 54 lower extremities in 46 patients scheduled for 1470-nm endovenous laser ablation under local anesthesia to treat CVI in a tertiary hospital. Patients were evaluated preoperatively, intraoperatively, and postoperatively over 30 days with clinical examination, physical examination, and ultrasound.

**Results:**

In the 54 lower extremities treated, there was a significant difference (p < 0.003) in terms of reduction in the diameter of treated veins (6.37 mm preoperatively and 5.15 mm on the 30th postoperative day) and improvement in the venous clinical severity score (VCSS) (means of 8.02 preoperative and 6.11 on the 30th postoperative day) (95%CI, 5.01—7.21) (p < 0.02). Postoperative complications such as paresthesia and phlebitis were present and diagnosed in 5 and 3 patients, respectively, but did not affect their quality of life or routine activities.

**Conclusions:**

Intravenous laser ablation of the SSV proved to be safe and effective for reducing clinical symptoms and improving quality of life.

## INTRODUCTION

Chronic venous insufficiency (CVI) is characterized by signs and symptoms produced by venous hypertension, primarily in the lower limbs. Symptoms frequently described by patients are pain, feelings of heaviness in the legs, cramps, itching, and edema. Signs that may be present depending on the stage of disease progression are cutaneous hyperpigmentation, rarefaction of hair, lipodermatosclerosis, eczema, and ulcerations.^1^

The prevalence of CVI is 25 to 33% among women and 10 to 20% among men in the Western adult population.^2^ This pathology has a direct impact on the working population, which increases its burden on public expenditure, since it is the 16th ranked cause of sick leave from work in Brazil.^3^

Clinical treatment for CVI involves medications, elastic compression, adoption of a healthy diet, and physical exercises. Over recent decades, new surgical and sclerotherapy treatment options have emerged for varicose veins.^1^ Endovenous laser thermoablation (EVLT) of saphenous veins is a minimally invasive technique that has been growing in use over the last 20 years and which achieves similar medium-term results to conventional surgical treatments.^4^

Therefore, in view of the increasing use and appreciation of EVLT as a method that yields better results than the conventional surgical technique for treatment of CVI, this study assessed patients who had small saphenous veins treated with the endolaser technique and were followed up for 30 days after surgery.

The primary objective was to assess the efficacy of CVI treatment in patients who had small saphenous vein (SSV) treatment with endolaser. The secondary objective was to assess post-surgical symptomology and complications during a 30-day period after the procedure.

## METHODS

The study is based on a longitudinal, retrospective analysis of patients diagnosed with chronic venous insufficiency due to SSV reflux who underwent thermoablation treatment under local anesthesia with a laser with a wavelength of 1470 nm, registered with the Brazilian National Health Surveillance Agency (ANVISA, 80058580018), using either radial or linear emission 600 μm fibers. All patients were treated surgically at a single center and the database was populated prospectively (a longitudinal protocol study) from June 2016 to September 2019. The project was approved by the Ethics Committee at our institution, ruling number 2.410.012.

Symptomatic patients with abnormalities on ultrasound were assessed for surgical treatment individually.

### Inclusion criteria

Patients were included who had symptoms compatible with CVI and were treated with thermoablation of the SSV, for whom full clinical and imaging assessments were available from 30-day outpatients follow-up.

### Exclusion criteria

Patients were excluded from thermoablative treatment if the SSV was less than 5 mm from the skin and/or tortuous, if medical record data were incomplete, or if they were treated surgically under spinal anesthesia.

The individuals included underwent clinical and echographic control assessments, as follows:

- Pre-surgical: chronic venous disease classification with Clinical, Etiology, Anatomy, Pathophysiology (CEAP) staging, venous clinical severity score (VCSS), age, sex, body mass index (BMI), and vein diameter at the saphenopopliteal junction (SPJ);
- Intraoperative: length of SSV treated, type of laser fiber used, linear energy endoluminal density (LEED), difficulties advancing the fiber up the SSV, flow on Doppler ultrasonography after thermoablation, surgical treatment of the great saphenous vein (GSV) during the same session, and perioperative pain;
- During the postoperative period, follow-up consultations were conducted from day 3 to day 5 and on the 30th postoperative day, assessing: SSV obliteration rate and absence of reflux, SPJ diameter, postoperative pain, number of analgesic pills taken, ecchymosis, paresthesia, phlebitis, presence of deep venous thrombosis (DVT) in the limb treated, and repeat VCSS assessment. The postoperative assessment was conducted using the same equipment, but due to differences in patients’ and surgeons’ agendas, follow-up consultations were conducted by different physicians.

Echographic assessment of the SPJ was conducted according to the classification described in the clinical practice guidelines for management of patients with varicose veins and venous diseases published by the Society for Vascular Surgery and the American Venous Forum. Absence of patent stump, partially patent stump, and absence of reflux were assessed. Treatment was defined as satisfactory if a patent stump and reflux were both absent (Table 1).^4^

**Table 1:** Proposal for classification of the results of Doppler ultrasonography of the saphenopopliteal junction after thermal ablation.

	J0	Absence of patent stump
**Patency**		
	J1, J2, J3, J4 etc.	Junction with patent stump length of 1, 2, 3, 4 cm etc.
	R+	Reflux
**Reflux**		
	R-	No reflux

J1: saphenopopliteal junction 1 cm in length, J2: saphenopopliteal junction 2 cm in length. J3: saphenopopliteal junction 3 cm in length, J4: saphenopopliteal junction 4 cm in length.

The procedures were conducted under local anesthesia in a surgical center with the patient in the prone position. The SSV to be treated was identified with intraoperative Doppler ultrasonography and then a bleb of anesthetic was injected into the chosen puncture site. Next, under Doppler ultrasound guidance, the SSV was punctured with an Abocath®16, preferably at a distal point after the last tributary vein with reflux, and then a 6Fr valved introducer was fitted. The endolaser fiber was positioned 2.5 cm from the SPJ, local tumescent anesthetic induction was administered along the length of the vein segment to be treated, using a syringe containing a solution made up from 250 mL of 0.9% saline, 20 mL of 2% lidocaine and adrenaline 1:100,000, and 4 mL of 8.4% sodium bicarbonate. After induction of anesthesia, the position of the fiber tip 2.5 cm from the SPJ was confirmed and the laser was activated. The laser was set to discharge 6 watts of power. During the operation, the fiber was manually tractioned continuously in the caudal direction all the way to the distal end of the small saphenous, beyond the largest caliber tributary vein.

Once thermal ablation was complete, extrinsic compression was applied along the path of the SSV using cotton pads and mid-thigh medium compression stockings (20-30 mmHg). Patients were discharged the same day, approximately 2 hours after surgery, and were encouraged to walk, but to remain relatively rested, returning to their normal activities slowly and progressively. At discharge, they were prescribed analgesics and nonsteroidal anti-inflammatories for 5 days and instructed to take them as necessary. The elastic stockings and extrinsic compression were kept on for 48 hours after which period the patients removed them themselves. Up until day 30, they were instructed to wear the stockings during the day and remove them for bathing and at night.

Results are presented using descriptive statistics. Quantitative variables are expressed using mean, median, minimum and maximum values, first and third quartiles, and standard deviation. Quantitative variables are compared between two different assessments using Student’s *t* test for paired samples and the Wilcoxon nonparametric test. P values smaller than 0.05 are indicative of statistical significance.

## RESULTS

A total of 46 patients were enrolled on the study, 12 of whom were male (26.08%) and 34 of whom were female (73.91%). The mean of age of these patients was 58.1 years (standard deviation [SD] 11.6 years; minimum 33 years; maximum, 84 years). Mean BMI was 29.6 (SD 4.24; minimum 21.9; maximum 39.6).

Eight of these 46 patients had both small saphenous veins treated in the same session, with a total of 54 lower limbs treated, 40 lower limbs in women and 14 lower limbs in men. Eight of the 54 limbs treated were CEAP stage C2, 18 were C3, 11 were C4, nine were C5, and eight limbs were CEAP stage C6. With regard to VCSS, the patients’ mean score before treatment was 8.02 (SD 5.19; maximum 24; minimum 2). At the first follow-up, the mean score had fallen to 7.26, and at the 30-day follow-up the mean score had fallen further, to 6.11 (95%CI 5.01-7.21). This reduction was statistically significant (Table 2).

**Table 2:** Comparison of diameters of small saphenous veins and change in venous clinical severity score at different times.

**Variable**	**Time**	**n**	**Mean**	**Minimum**	**Maximum**	***p*-value**
	Preoperative	54	6.37	2	13	
Diameter (mm)	3-5 days	54	4.99	1.5	10.6	< 0.003
	30th preoperative	54	5.15	1.4	10	
	day	54	8.02	2	24	
VCSS	3-5 days	54	7.26	2	20	< 0.02
	30th day	54	6.11	1	19	

n = lower limbs treated; p-value equal or less than 0.05 is statistically significant.

Ultrasound-guided puncture was the technique used to insert the 1470 nm/600 μm fiber in all procedures. In 37 (68.51%) of the limbs treated, a two-ring radial fiber was used and a linear fiber was used in the remaining 17 (31.48%). Mean LEED was 63.49 J/cm (SD 10.45 J/cm; minimum 41.6 J/cm; maximum 85 J/cm).

The length of the segment treated, measured from the SPJ, ranged from 4 to 31 cm (SD 5.62 cm), with a mean length of 17.26 cm treated. In turn, the diameter of the small saphenous veins before treatment ranged from 2 mm to 13 mm (SD 2.41 mm), with a mean of 6.37 mm. On day 30, mean diameter was 5.15 (95%CI 4.58-5.72). The change in venous diameter over the three assessments can be observed in Table 2. The rate of small saphenous occlusion was 100% in the immediate postoperative period, 98.15% at 3 to 5 days, and 96.30% at 30 days.

With regard to complications inherent to the surgical treatment, none of our patients was diagnosed with deep venous thrombosis by ultrasound. At the first follow-up, 23 of the treated limbs had ecchymosis. At the 30-day follow-up, just one of these limbs still had signs of ecchymosis, with no clinical significance for the patient. With regard to phlebitis, at the first follow-up three of the lower limbs treated were diagnosed with thrombophlebitis and the same three patients still complained of phlebitis at the 30-day follow-up, although this had not prevented them from resuming their usual activities.

When asked about use of analgesics after surgical treatment, 11 of the 46 patients operated stated that they had needed oral analgesia to control pain during the first 5 days. At 30 days, only two patients were still taking analgesics. Paresthesia was assessed verbally and by physical examination and was diagnosed in seven patients at the first follow-up. At 30-day follow-up, five patients still had this complaint (Table 3).

**Table 3:** Complications related to the procedure.

	n (%)	3-5 days	Day 30
DVT	54 (100%)	0 (0%)	0 (0%)
Paresthesia	54 (100%)	7 (12.96%)	5 (9.26%)
Reflux	54 (100%)	1 (1.85%)	2 (3.7%)
Phlebitis	54 (100%)	3 (5.55%)	3 (5.55%)
Ecchymosis	54 (100%)	23 (42.59%)	1 (1.85%)

n = lower limbs treated; DVT = deep venous thrombosis.

## DISCUSSION

Statistically, the SSV is involved in 15% of chronic venous insufficiency cases.^5^ However, reflux through this vein is almost always highly symptomatic and can be responsible for trophic changes and ulcers, making treatment more difficult.^6^

Anatomically, the SSV emerges from a junction between veins that ascend from the lateral extremity of the dorsal venous arch of the foot and the lateral marginal vein, running along the posterior lateral malleolus of the tibia. It ascends within the saphenous compartment, lateral to the calcaneus tendon, accompanied by the sural nerve up to the popliteal fossa. In his doctoral thesis, Burihan reported that the SSV enters the fascia of the leg from 10 to 20 cm above the intermalleolar line in 62.5% of cases and is entirely subcutaneous in just 2.05% of cases.^7^ The saphenous compartment ceases to exist at the popliteal fossa because the muscular fascia adheres to the fascia of the gastrocnemius muscles. From this point upwards, there are several variant SSV paths, which have been the object of several studies over recent decades. Communication between the small and great saphenous veins is a very common variant, variously known as the femoropopliteal vein, posterior femoral cutaneous vein, anastomotic superior branch, or Giacomini vein. In this variant, the upper third of the SSV communicates with the GSV at the transition from the mid third to the upper third; but it can even communicate with the GSV close to the saphenofemoral junction.^7^ Early studies were conducted with cadavers, but more recent studies employ Doppler ultrasonography.^8^

**Figure 1:**
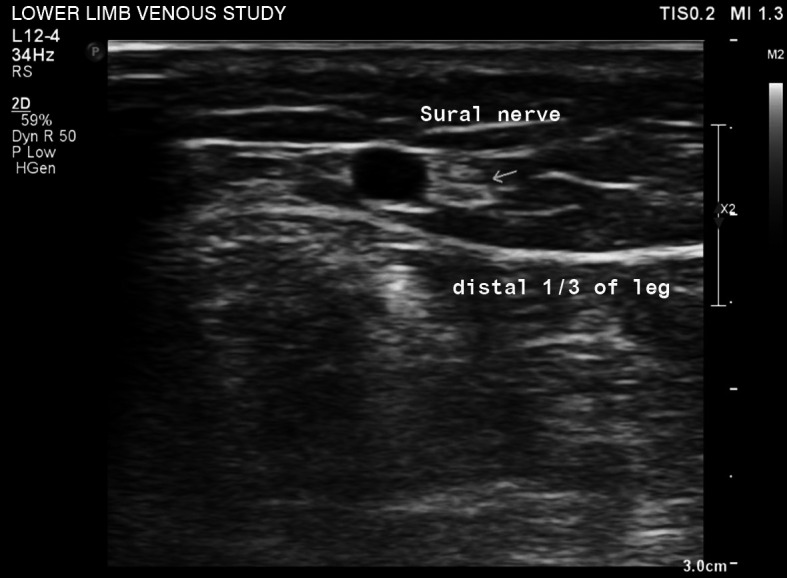
Echographic image in mode B showing the small saphenous vein in the distal 1/3 of the leg and its proximity to the sural nerve.

In the recent past, conventional surgery was the technique of choice for treatment of SSV insufficiency. With regard to technique, Telling et al.^5^ demonstrated that 75.7% of surgeons simply performed ligature of the small saphenous vein as close as possible to the saphenopopliteal junction, while 14.5% routinely stripped the vein. Injury to the sural nerve is a complication of the conventional technique that merits significant concern (Figure 1).

With the advent of EVLT treatment, neurological sural injuries were reduced substantially. This is primarily due to use of anesthetic solution for tumescence. Studies show that tumescence protects the perivascular structures (nerves and skin), primarily by dissipating the heat generated by the endovenous laser and also because it reduces the diameter of the vessel being treated, enabling the target vessel to better absorb the heat.^9^

The length of SSV treated in the present study (mean of 17.26 cm; SD 5.62 cm) is close to the length treated by Nwaejike (18 cm)^10^ and Theivacumar (17 cm).^11^ The mean diameter of the veins treated was 6.37 mm, which is comparable to the mean diameters in a series by Elias and Khilnani^12^ (50 limbs and mean diameter of 5.8 mm) and in the patients in the study by Theivacumar (mean diameter was 6.2 mm in 68 limbs treated). In our analysis, there was a significant reduction in SSV diameter after treatment, with initial mean diameter of 6.37 mm reducing to 5.15 mm (95%CI 4.58-5.72) at 30-day follow-up (p < 0.003) (Table 2). This reduction was explained by Heger et al. in terms of the theory of late inflammatory response, according to which the thrombus created by the laser heat in the blood would later release a series of inflammatory mediators, attracting remodeling cells such as fibroblasts and macrophages. This inflammatory reaction would cause fibrosis and, later, venous occlusion.^13^

Our analysis of the complications of surgical treatment for varicose veins showed that paresthesia was reported in 13% of the patients treated at the 3-to-5-day follow-up, falling to 9.3% at 30 days’ follow-up. Desmyttère et al.^14^ published a study in which they followed 128 patients who underwent thermoablation of the small saphenous for 3 years, reporting that 40% of the sample exhibited paresthesia at 15 days’ follow-up, but that this symptom was not reported after 30 days’ follow-up.

The VCSS analysis revealed that the there was a statistically significant reduction in this score. Mean score at baseline was 8.02, reducing to 7.26 at the first follow-up and 6.11 (95%CI 5.01-7.21) at the 30-day follow-up (p < 0.02) (Table 2). In contrast with studies of the great saphenous vein, few studies have reported change in VCSS after SSV treatment. In 2007, Theivacumar et al. published a study assessing clinical improvement with the Aberdeen Varicose Vein Symptom Severity Score (AVSS), showing significant improvement in AVSS.^10^

It is known that patient age, clinical severity of chronic venous disease, and a hypercoagulable state are all factors that increase the risk of DVT in thermoablative procedures. In this study, the results were similar to those of other articles about the small saphenous vein.^15^^,^^16^ None of the patients had DVT during follow-up.

During routine follow-up, we observed that just one patient out of the 54 lower limbs treated in the study (1.85%) had saphenous flow at the first postoperative follow-up and four patients (7.4%) had flow on control Doppler ultrasonography at the 30 day postoperative follow-up. In 2016, Boersma et al.^17^ analyzed 2,950 patients treated with endolaser of the small saphenous, reporting an SSV occlusion rate of 98.5% (95%CI 97.7-99.2). In 2007, Kathleen D. Gibson et al.^18^ published a prospective study in which 126 patients were followed for 6 months after treatment with endolaser of the small saphenous, observing complete saphenous occlusion in 96% of cases.

With relation to other treatment techniques, we searched for studies that compared the conventional technique with EVLT. The European Society for Vascular Surgery clinical practice guidelines for management of chronic venous disease^19^ state that thermal ablation of the small saphenous is more effective and causes fewer side effects than other techniques. In 2013, Samuel et al.^20^ randomized 106 patients with unilateral reflux of the small saphenous into two groups of 53 people. One group was treated with the conventional technique and the other group was treated with EVLT. After follow-up at 1, 6, 12, and 52 weeks, they observed that EVLT eliminated reflux in 96.2% of cases, compared to 71.7% for the conventional technique. There was also a lower rate of paresthesia (7.5% for EVLT vs. 26.4% for the conventional technique). They therefore concluded that the clinical benefits of both techniques are similar, but that EVLT was more effective for treatment of the underlying pathophysiology and was associated with lower perioperative morbidity, enabling faster recovery. ^Boersma et al.^ ^17^ published a systematic review that analyzed 49 articles (five randomized clinical trials and 44 cohort studies) comparing the different techniques for treatment of CVI of the small saphenous. The review concluded that EVLT has better primary and secondary success rates than both the conventional technique and sclerotherapy.

One limitation of this study was the absence of patients treated surgically under spinal anesthesia. We excluded this subset, because when patients were treated using this type of anesthetic induction, we performed thermoablation and varicectomy in the same operation. We observed that this feature introduced a confounding factor for patients, since there was the necessary differentiation between the pain and paresthesia symptoms of varicectomy or thermoablation of the small saphenous vein was lacking during outpatient follow-up, especially in vessels close to the saphenous path. Other limitations include the small and heterogeneous sample, with patient age varying from 33 to 84 years and a range of different CEAP stages. In this study we were unable to assess patients beyond 30 days, since few patients return to the clinic after this period, preventing collection of the data needed for statistical analysis. We also did not reassess patients for CEAP stage at postoperative follow-up. This article nevertheless reports similar results to other studies, even those with longer follow-up and larger samples.

## CONCLUSIONS

The laser thermoablation of the SSV technique has important advantages for treatment choice, making it an important therapeutic option. Ablative therapy proved safe and effective for reducing clinical symptoms and improving quality of life. Studies with long-term follow-up would be able to confirm these claims, further establishing thermoablation as a treatment for the small saphenous vein.
