# Supplementary material for: Evaluation of endolaser thermoablation of the small saphenous vein under local anesthesia
Source: J Vasc Bras. 2021 Aug 2;20:e20200215. doi: 10.1590/1677-5449.200215 (PMC8336979; doi:10.1590/1677-5449.200215)
Supplement: Translation

# Translation: Avaliação da termoablação com endolaser da veia safena parva com anestesia local

**Authors:** Filipe Cézar Bertassoni de Souza, Walter Jr. Boim de Araujo, Adriana Buechner de Freitas Brandao, Camila de Almeida Mazzoni, Fabiano Luiz Erzinger, Filipe Carlos Caron, Viviane Gomes Milgioransa Ruggeri

**Affiliations:** 1 Hospital Angelina Caron, Campina Grande do Sul, PR, Brasil.; 2 Instituto da Circulação - Excelência em Angiologia, Cirurgia Vascular e Endovascular, Curitiba, PR, Brasil.

**Keywords:** varizes, termoablação por endolaser, insuficiência venosa crônica, veia safena parva, anestesia local

## Abstract

**Contexto:**

Na insuficiência venosa crônica (IVC), a veia safena parva (VSP) é afetada em 15% dos casos. A cirurgia convencional é a técnica padrão para o tratamento da insuficiência da VSP, sendo a lesão no nervo sural uma complicação bastante temida. O tratamento de termoablação com endolaser tende a ser um método cirúrgico que diminui complicações da terapia cirúrgica da IVC.

**Objetivos:**

Avaliar os pacientes com IVC submetidos à terapia por endolaser da VSP ao menos 30 dias após o procedimento.

**Métodos:**

Foram analisados 54 membros inferiores de 46 pacientes submetidos à terapia por endolaser 1470 nm, sob anestesia local, para o tratamento da IVC em um hospital terciário. Os pacientes foram avaliados no período pré-operatório, intraoperatório e pós-operatório de 30 dias, através da clínica, exame físico e achados ecográficos.

**Resultados:**

Nos 54 membros inferiores submetidos ao tratamento, comparando-se o período pré-operatório e o 30º dia pós-operatório, houve diferença significativa (p < 0,003) na redução do diâmetro da VSP tratada (6,37 mm pré-operatório e 5,15 mm no 30º dia pós-operatório) (IC95% 4,58-5,72) e na melhora do escore de gravidade clínica venosa (VCSS) (média de 8,02 pré-operatório e 6,11 no 30º dia pós-operatório) (IC95% 5,01-7,21) (p < 0,02). Complicações pós-operatórias, como parestesia e flebite, estiveram presentes e foram diagnosticadas em cinco e três pacientes, respectivamente, sem significar alteração na qualidade de vida e nas atividades de rotina.

**Conclusões:**

A técnica de termoablação com *laser* da VSP mostrou-se segura e eficaz na redução dos sintomas clínicos e na melhora da qualidade de vida.

## INTRODUÇÃO

A insuficiência venosa crônica (IVC) é caracterizada por sinais e sintomas produzidos pela hipertensão venosa, principalmente nos membros inferiores. Dor, sensação de peso nas pernas, câimbras, pruridos e edemas são sintomas comumente relatados pelo paciente. Hiperpigmentação cutânea, rarefação de pelos, lipodermatoesclerose, eczema e ulcerações são sinais presentes, dependendo da fase de evolução da doença^1^.

A IVC possui uma prevalência de 25 a 33% das mulheres e 10 a 20% dos homens na população adulta ocidental^2^. Assim, essa patologia influencia diretamente nas atividades laborais, onerando ainda mais os gastos públicos, sendo a 16ª causa de afastamento do trabalho no Brasil^3^.

O tratamento clínico da IVC envolve uso de medicamentos, compressão elástica, medidas higienodietéticas e prática de exercícios físicos. Nas últimas décadas, surgiram novas opções terapêuticas cirúrgicas e escleroterápicas para o tratamento das varizes^1^. A termoablação endovenosa das safenas através do endolaser (EVLT) é umas das técnicas minimamente invasivas, crescente nos últimos 20 anos, apresentando resultados semelhantes a médio prazo ao da terapia cirúrgica convencional^4^.

Portanto, pela crescente utilização e valorização da EVLT como um método que apresenta resultados melhores do que a técnica cirúrgica convencional do tratamento da IVC, este trabalho visa avaliar os pacientes tratados pela técnica de endolaser de safenas parvas, que realizaram acompanhamento pós-cirúrgico de 30 dias.

O objetivo primário foi avaliar a eficácia do tratamento da IVC nos pacientes submetidos ao tratamento com endolaser da veia safena parva (VSP). O objetivo secundário foi avaliar a sintomatologia pós-cirúrgica e complicações após o procedimento, no período de 30 dias.

## MÉTODOS

Trata-se de uma análise longitudinal, retrospectiva, de pacientes diagnosticados com insuficiência venosa crônica devido ao refluxo da VSP, submetidos à terapia termoablativa, com *laser* de comprimento de onda 1470 nm, registrado na Agência Nacional da Vigilância Sanitária (80058580018), utilizando fibras de 600 μm, de emissão radial ou linear, sob anestesia local. São pacientes tratados cirurgicamente em um único centro, tendo como base o banco de dados alimentado prospectivamente (estudo longitudinal sob protocolo) no período de junho 2016 a setembro de 2019. O projeto foi aprovado pelo Comitê de Ética da nossa instituição, sob o parecer nº 2.410.012.

Foram avaliados para o tratamento cirúrgico pacientes sintomáticos e com alteração ecográfica individualmente.

### Critérios de inclusão

Foram incluídos doentes com sintomas compatíveis com IVC, tratados por técnica termoablativa da VSP, que apresentavam avaliação clínica completa e de imagem no acompanhamento ambulatorial de seguimento por 30 dias.

### Critérios de exclusão

Foram excluídos do tratamento termoablativo pacientes com VSP com menos de 5 mm de distância da pele e/ou tortuosa, com dados incompletos nos prontuários e tratados cirurgicamente sob raquianestesia.

Os indivíduos foram avaliados através do controle clínico e ecográfico da seguinte forma:

- Pré-cirúrgico: classificação para as doenças venosas crônicas, baseada na Clínica, Etiologia, Anatomia e Fisiopatologia (CEAP), escore da gravidade clínica venosa (VCSS), idade, sexo, índice de massa corpórea (IMC), diâmetro da junção safeno-poplítea (JSP);
- Intraoperatório: extensão tratada da VSP, tipo de fibra do *laser*, quantidade da densidade da energia endoluminal linear (LEED), dificuldade de subir a fibra na VSP, fluxo ao eco-Doppler após termoablação, tratamento no mesmo tempo cirúrgico da veia safena magna (VSM) e dor perioperatória;
- No pós-operatório, houve acompanhamento do 3º ao 5º e no 30º dia pós-operatório, sendo avaliados: taxa de obliteração da VSP e ausência de refluxo, diâmetro da JSP, dor pós-operatória, quantidade de comprimidos analgésicos utilizados, equimose, parestesia, flebite, presença de trombose venosa profunda (TVP) no membro tratado e novamente o VCSS. Para a avalição pós-operatória, utilizamos o mesmo aparelho. Entretanto, devido a diferenças na agenda do paciente e dos cirurgiões, o retorno foi avaliado por outros médicos.

Ecograficamente, a avaliação da JSP seguiu a classificação descrita nas diretrizes de prática clínica para o manejo de pacientes com varizes e doenças venosas da Society for Vascular Surgery e da American Venous Forum. Avaliou-se a ausência de coto pérvio, coto parcialmente pérvio e a ausência de refluxo. Considerou-se como tratamento satisfatório a ausência de coto pérvio e de refluxo (Tabela 1)^4^.

**Tabela 1:** Proposta de classificação dos resultados do eco-Doppler na junção safeno- poplítea após a termoablação.

	J0	Ausência de coto pérvio
**Perviedade**		
	J1, J2, J3, J4 etc.	Junção com coto pérvio de 1, 2, 3, 4 cm etc.
	R+	Refluxo
**Refluxo**		
	R-	Sem refluxo

J1: junção safeno-poplítea 1 cm extensão, J2: junção safeno-poplítea 2 cm extensão. J3: junção safeno-poplítea 3 cm extensão, J4: junção safeno-poplítea 4 cm extensão.

O procedimento, praticado com anestesia local, foi realizado em centro cirúrgico, com o paciente em decúbito ventral. Identificava-se a VSP a ser tratada, através do eco-Doppler intraoperatório e então efetuava-se um botão anestésico no local da punção escolhida. Após, guiado pelo eco-Doppler, puncionava-se a VSP com Abocath®16, preferencialmente em um ponto distal após a última veia tributária com refluxo, seguido da instalação do introdutor valvulado 6F. Após o posicionamento da fibra de endolaser distando 2,5 cm da JSP, realizava-se a indução anestésica local tumescente ao longo de todo o trajeto a ser tratado. Utilizava-se seringa com uma solução composta por 250 mL de solução fisiológica a 0,9%, 20 mL de lidocaína a 2% e adrenalina 1:100.000 com 4 mL de bicarbonato de sódio a 8,4%. Após a anestesia, conferia-se novamente a posição da fibra a 2,5 cm da JSP e iniciava-se o disparo do *laser*. O aparelho estava configurado para disparar uma potência de 6 watts. Durante a cirurgia, tracionava-se a fibra manualmente e de forma contínua em direção caudal até o limite distal da safena parva após a veia tributária mais calibrosa.

Terminada a termoablação, confeccionava-se compressão extrínseca no trajeto da VSP com chumaço de algodão e meia elástica 7/8 de média compressão (20-30 mmHg). Os pacientes tratados receberam alta no mesmo dia, aproximadamente 2 horas após a cirurgia, sendo estimulados a deambular e orientados a fazer repouso relativo, voltando ao retorno de suas atividades normais, lenta e progressivamente. Na alta, foram prescritos analgésicos e anti-inflamatórios não esteroidais por 5 dias e os pacientes foram orientados a utilizá-los conforme a necessidade. A meia elástica e a compressão extrínseca eram mantidas durante 48 horas; a meia era retirada pelo próprio paciente. Até o 30º dia, orientava-se a manutenção da meia elástica durante o dia e sua retirada para tomar banho e para dormir.

Os resultados foram expressos através da estatística descritiva. Para descrição das variáveis quantitativas foram consideradas as estatísticas de média, mediana, valor mínimo, valor máximo, 1º e 3º quartis e desvio padrão. Para comparação de dois momentos de avaliação em relação a variáveis quantitativas foram considerados os testes *t* de Student para amostras pareadas e não paramétrico de Wilcoxon. Valores de p menores do que 0,05 indicaram significância estatística.

## RESULTADOS

Ao todo, 46 pacientes foram incluídos no estudo, sendo 12 do sexo masculino (26,08%) e 34 do sexo feminino (73,91%). A média de idade dos pacientes era de 58,1 anos [desvio padrão (DP) 11,6 anos; mínimo 33 anos; máximo, 84 anos]. O IMC médio era de 29,6 (DP 4,24; mínimo de 21,9 e máximo de 39,6).

Desses 46 pacientes, oito trataram no mesmo tempo cirúrgico ambas as safenas parvas, totalizando assim 54 membros inferiores tratados, sendo 40 membros inferiores em mulheres e 14 membros inferiores em homens. Dos 54 membros tratados, oito membros eram da classe clínica CEAP C2, 18 eram C3, 11 eram C4, nove membros eram C5 e oito eram C6. Em relação ao VCSS, a média relatada pelos pacientes antes do tratamento foi de 8,02 (DP 5,19, máximo 24 e mínimo de 2); no primeiro retorno, a média foi reduzida para 7,26 e no retorno do 30º dia a média reduziu para 6,11 (IC95% 5,01-7,21). Essa redução mostrou-se significativa estatisticamente (Tabela 2).

**Tabela 2:** Comparação dos diâmetros da veia safena parva e da evolução do escore de gravidade clínica venosa em diferentes momentos.

**Variável**	**Tempo**	**n**	**Média**	**Mínimo**	**Máximo**	**p-valor**
	Pré-operatório	54	6,37	2	13	
Diâmetro (mm)	3-5 dias	54	4,99	1,5	10,6	< 0,003
	30º dia	54	5,15	1,4	10	
	Pré-operatório	54	8,02	2	24	
VCSS	3-5 dias	54	7,26	2	20	< 0,02
	30º dia	54	6,11	1	19	

n = membros inferiores tratados; valor de p significativo quando igual ou menor que 0,05.

A punção ecoguiada foi a técnica utilizada em todos nossos procedimentos para a inserção da fibra 1470 nm / 600 μm. Em 37 (68,51%) membros tratados, utilizou-se a fibra radial com dois anéis e em 17 (31,48%) utilizou-se a fibra tipo linear. O LEED médio foi de 63,49 J/cm (DP 10,45 J/cm, mínimo de 41,6 J/cm e máximo de 85 J/cm).

A extensão, desde a croça da VSP tratada, variou de 4 a 31 cm (DP 5,62 cm), tendo uma média de 17,26 cm tratados. Em relação ao diâmetro das safenas parvas, houve variação de 2 mm a 13 mm (DP 2,41 mm), com uma média de 6,37 mm antes do tratamento. No 30º dia, a média do diâmetro foi de 5,15 (IC95% 4,58-5,72). A evolução da média do diâmetro venoso pode ser avaliada na Tabela 2. A taxa de oclusão da safena parva foi de 100% no pós-operatório imediato, 98,15% em 3 a 5 dias e 96,30% em 30 dias.

Das complicações inerentes ao tratamento cirúrgico, nenhum dos nossos pacientes foi diagnosticado através de exame ecográfico com trombose venosa profunda. No primeiro retorno, foram relatados 23 membros tratados com equimose. No 30º dia do acompanhamento, apenas um membro manteve o sinal equimótico, sem mostrar significância clínica ao paciente. Em relação à queixa de flebite, no primeiro retorno três membros inferiores tratados foram diagnosticados com tromboflebite e esses mesmos pacientes mantiveram a queixa de flebite no retorno de 30 dias, sem limitá-los ao retorno às atividades rotineiras.

Quando indagados sobre o uso de analgésicos após o tratamento cirúrgico, dos 46 pacientes operados, 11 necessitaram de analgesia via oral para controle da dor nos primeiros 5 dias. No segundo retorno (30 dias), apenas dois pacientes mantiveram o uso de analgésicos. A parestesia foi avaliada pela anamnese e exame físico e relatada em sete pacientes no primeiro retorno. No retorno do 30º dia pós-operatório, cinco pacientes mantiveram essa queixa (Tabela 3).

**Tabela 3:** Complicações relacionadas ao procedimento.

	n (%)	3-5 dias	30º dia
TVP	54 (100%)	0 (0%)	0 (0%)
Parestesia	54 (100%)	7 (12,96%)	5 (9,26%)
Refluxo	54 (100%)	1 (1,85%)	2 (3,7%)
Flebite	54 (100%)	3 (5,55%)	3 (5,55%)
Equimose	54 (100%)	23 (42,59%)	1 (1,85%)

n = membros inferiores tratados; TVP = trombose venosa profunda.

## DISCUSSÃO

Na insuficiência venosa crônica, estatisticamente a VSP é afetada em 15% das vezes^5^. Todavia, a presença do refluxo dessa veia quase sempre é bastante sintomática, podendo ser responsável por alterações tróficas e úlceras, dificultando o tratamento^6^.

Anatomicamente, a VSP surge da junção de veias que ascendem a partir da extremidade lateral do arco venoso dorsal com a veia marginal lateral do pé, percorrendo a região posterior do maléolo lateral da tíbia. Ascende pelo compartimento safênico, lateralmente ao tendão calcâneo, acompanhada pelo nervo sural até a fossa poplítea. Burihan, em sua tese de doutorado, relatou que a VSP adentra a fáscia da perna entre 10 e 20 cm acima da linha intermaleolar em 62,5% dos casos e em somente 2,05% ela se apresenta totalmente subcutânea^7^. No cavo poplíteo, o compartimento safênico deixa de existir, devido à adesão da fáscia muscular às fáscias dos músculos gastrocnêmios. A partir desse ponto, a continuidade da VSP possui diversas variações, sendo objeto de diversos estudos nas últimas décadas. Uma comunicação entre a safena parva e a safena magna é uma variante bastante comum e chamada de veia fêmoro-poplítea, veia cutâneo femoral posterior, ramo anastomótico superior ou veia de Giacomini. Essa variante comunica o terço superior da VSP com a VSM na transição do terço médio com o superior; porém, pode se comunicar com a VSM até próximo da junção safeno-femoral^7^. No início, estudos eram realizados em cadáveres e, mais recentemente, através do uso do eco-Doppler^8^.

Em um passado recente, a cirurgia convencional era a técnica de escolha para o tratamento da insuficiência da VSP. Sobre essa técnica, Telling SS et al.^5^ demonstraram que 75,7% dos cirurgiões realizam apenas a ligadura da safena parva o mais próximo possível da junção safeno-poplítea e 14,5% realizam rotineiramente a fleboextração. Utilizando a técnica convencional, a lesão do nervo sural é uma complicação bastante temida (Figura 1).

**Figura 1:** Imagem ecográfica em modo B evidenciando a veia safena parva no 1/3 distal da perna e sua proximidade com o nervo sural.

Com o advento do tratamento através da EVLT, a lesão neurológica sural diminuiu substancialmente. Isso se deve principalmente à utilização da solução anestésica para intumescência. Estudos mostram que a intumescência protege as estruturas perivasculares (nervos e pele), principalmente por dissipar o calor oriundo do *laser* endovenoso e por diminuir o diâmetro do vaso a ser tratado, permitindo uma melhor absorção do calor no vaso alvo^9^.

No presente estudo, a extensão de veia parva tratada (média de 17,26 cm, DP 5,62 cm) é próxima da tratada por Nwaejike (18 cm)^10^ e por Theivacumar (17 cm)^11^. O diâmetro médio venoso tratado foi de 6,37 mm, comparável aos diâmetros médios tratados nas séries de Elias e Khilnani^12^ (50 membros com média de 5,8 mm) e dos pacientes do estudo de Theivacumar (em 68 membros tratados, o diâmetro médio foi de 6,2 mm). Em nossa análise, houve redução significativa no diâmetro da VSP tratada, com média inicial de 6,37 mm, reduzindo para 5,15 mm (IC95% 4,58-5,72) no retorno do 30º dia (p < 0,003) (Tabela 2). Essa redução foi explicada por Heger et al. através da teoria da Resposta Inflamatória Tardia, em que o trombo gerado pelo calor do *laser* no sangue liberaria tardiamente uma série de mediadores inflamatórios, atraindo células cicatriciais, como fibroblastos e macrófagos. Essa reação inflamatória resultaria em fibrose e tardiamente na oclusão venosa^13^.

Quando analisamos as complicações do tratamento cirúrgico de varizes, a parestesia foi relatada em 13% dos pacientes submetidos ao tratamento, no período entre 3º e o 5º dia, caindo para 9,3% no 30º dia de acompanhamento. Desmyttère et al.^14^ apresentaram um estudo que acompanhou por 3 anos 128 pacientes submetidos à termoablação de safena parva, sendo que 40% da amostra apresentaram parestesia nos 15 dias de acompanhamento, sendo esse sintoma não mais relatado após 30 dias de acompanhamento.

Na avaliação do VCSS, verificamos a redução estatisticamente significativa desse escore. A média inicial da variável foi de 8,02, diminuindo para 7,26 no primeiro retorno e 6,11 (IC95% 5,01-7,21) no retorno do 30º dia (p < 0,02) (Tabela 2). Diferentemente dos estudos para safena magna, há poucos estudos que mostram a evolução do VCSS após o tratamento da VSP. Em 2007, Theivacumar et al. publicaram um estudo avaliando a melhora clínica através do *Aberdeen Varicose Vein Symptom Severity Score* (AVSS). Nessa análise, houve melhora significativa do AVSS^10^.

Sabe-se que a idade do paciente, gravidade clínica da doença venosa crônica e o estado de hipercoagulabilidade são fatores que aumentam o risco de TVP em procedimentos termoablativos. No estudo, os resultados foram semelhantes a resultados de outros artigos sobre a safena parva^15^^,^^16^. Nenhum paciente apresentou TVP durante o acompanhamento.

No acompanhamento de rotina, vimos que, dos 54 membros inferiores tratados no estudo, apenas um paciente (1,85%) apresentou fluxo safênico no primeiro retorno de pós-operatório e quatro pacientes (7,4%) apresentaram fluxo no eco-Doppler de controle do 30º dia pós-operatório. Em 2016, Boersma et al.^17^ analisaram 2.950 pacientes tratados com o endolaser na safena parva. O estudo verificou uma taxa de oclusão da VSP de 98,5% (IC95% 97.7-99.2). Em 2007, Kathleen D. Gibson et al.^18^ publicaram um estudo prospectivo, em que 126 pacientes foram acompanhados por 6 meses e após o tratamento com endolaser da safena parva, tendo uma completa oclusão safênica em 96% dos casos.

Em relação a outras técnicas, procuramos estudos em que houve a comparação entre a técnica convencional e a EVLT. As Diretrizes de Prática Clínica da Sociedade Europeia de Cirurgia Vascular sobre o tratamento da doença venosa crônica^19^ relatam que, comparada com outras técnicas, a termoablação da safena parva é mais eficiente e resulta em menos efeitos colaterais. Em 2013, Samuel et al.^20^ randomizaram 106 pacientes com refluxo unilateral da safena parva em dois grupos de 53 pessoas. Um grupo foi tratado pela técnica convencional e o outro grupo foi tratado com a EVLT. Após acompanhamento de 1, 6, 12 e 52 semanas, observou-se que a EVLT aboliu o refluxo em 96,2% dos casos, contra 71,7% na técnica convencional. Constatou-se também um menor índice de parestesia (7,5% na EVLT *versus* 26,4% na técnica convencional). Conclui-se então que os benefícios clínicos são semelhantes em ambas técnicas, porém a EVLT foi mais eficiente no tratamento da fisiopatologia subjacente e apresentou menor morbidade perioperatória, permitindo uma recuperação mais rápida. Na revisão sistemática de ^Boersma et al.^ ^17^ foram analisados 49 artigos (cinco ensaios clínicos randomizados e 44 estudos de coorte) comparando as diversas técnicas de tratamento da IVC da safena parva. A revisão concluiu que a EVLT apresenta taxas de sucesso primário e secundário melhores que a técnica convencional e que a escleroterapia.

Uma limitação do estudo foi a ausência de pacientes tratados cirurgicamente sob raquianestesia. Excluímos, pois nessa indução anestésica, realizamos conjuntamente a termoablação e a varicectomia. Assim, observamos que esse fato era um agente de confusão para os pacientes, já que no retorno ambulatorial não havia a diferenciação necessária entre os sintomas álgicos e os parestésicos da varicectomia ou da termoablação da safena parva, principalmente em vasos próximos ao trajeto safênico. Outras limitações foram uma amostra pequena e heterogênea, com pacientes variando de 33 a 84 anos e com diferentes CEAPs. Neste estudo, não conseguimos avaliar os pacientes além dos 30 dias, uma vez que após esse período poucos pacientes retornavam ao consultório, dificultando assim a coleta de dados para aferição estatística. Também não classificamos os pacientes quanto ao CEAP nos regressos pós-cirúrgicos. Apesar disso, este artigo apresentou resultados semelhantes a outros estudos, mesmo esses sendo mais longos e com amostragens maiores.

## CONCLUSÃO

A técnica da termoablação com laser da VSP ganhou uma importante vantagem na escolha terapêutica, sendo uma importante opção terapêutica. A terapia ablativa mostrou-se segura e eficaz na redução dos sintomas clínicos e na melhora da qualidade de vida. Estudos com acompanhamento de longo prazo poderão corroborar essas afirmações e sedimentar ainda mais a terapia termoblativa de safena parva.
